# Effects of UV-B and Water Deficit on Aroma Precursors in Grapes and Flavor Release during Wine Micro-Vinification and Consumption

**DOI:** 10.3390/foods11091336

**Published:** 2022-05-04

**Authors:** Yifan Zhu, Meng Sun, Roland Harrison, Brian Jordan, Glen Creasy, Rainer Hofmann

**Affiliations:** 1 College of Food Science and Technology, Yunnan Agricultural University, Kunming 650201, China; yifanzhuyy@163.com; 2 Centre for Viticulture and Oenology, Faculty of Agriculture and Life Sciences, Lincoln University, Christchurch 7674, New Zealand; roland.harrison@lincoln.ac.nz (R.H.); brian.jordan@lincoln.ac.nz (B.J.); rainer.hofmann@lincoln.ac.nz (R.H.); 3 University Engineering Research Center for Grape & Wine of Yunan Province, Yunnan Agricultural University, Kunming 650201, China; 4 Jiangsu Academy of Agricultural Sciences (JAAS), Nanjing 210014, China; 5 Sabrosia SAS, 30170 Pompignan, France; creasyg@gmail.com

**Keywords:** UV-B, water deficit, amino acids, aroma compounds, micro-vinification, Pinot noir

## Abstract

UV-B radiation and water availability can affect amino acids(AAs) concentration in berries, resulting in the evolution of aroma compounds during alcoholic fermentation. This study investigated the effects of UV-B exposure and water availability onwine aroma compounds in Pinot noir, focusing on the role of AAs in the process.Enhanced UV-B radiation significantly decreased total AA concentrations and most individual AAs inberries and wines, while water deficitincreased some individual AAsin wines. Higher alcohols, fatty acids, esters, monoterpenes, and C_13_-norisoprenoids were affected by UV-B interaction with water deficit in wines. These results suggested individual or combined UV-B exposure and water deficit had direct effects on fruit AAs, leading to significant differences in some wine aroma compounds.

## 1. Introduction

Amino acids in grapes have potential influences on wine aroma profiles. Amino acids serve as a major nitrogen storage compound and are critical subunits and precursors of some aroma compounds in grapes [1,2,3]. Environmental factors can induce grapevine defense mechanisms and influence nitrogen metabolism in grapes [4,5]. Therefore, amino acids profiles might be modified by environmental factors, such as UV-B and water deficit.

Yeast utilizes amino acids as a nitrogen source to form aroma-active compounds during alcoholic fermentation [6]. Most important wine volatile compounds are generated during alcoholic fermentation as yeast secondary metabolites. General fermentation kinetics and the formations of esters, higher alcohols, and volatile fatty acids are related to amino acids [7]. Thus, amino acids can act as aroma precursors to drive aroma compounds during fermentation [8].

Some research involving the addition of amino acidsto must have been used to support the relationship between amino acids and wine aroma compounds [9]. However, there are no studies about the relationship between amino acid consumption and aroma compound development. To understand how and to what extent the differences in grapes can be transported into wines is lacking. This study is to investigate the influence of UV-B and water deficit on amino acid profiles, and in turn, their effect on wine aroma-active compounds. Consequently, this knowledge could help predict the relationship between amino acids and wine aroma compounds, which could help predict wine qualities based on pre-fermentation analysis of grapes.

## 2. Materials and Methods

### 2.1. Treatments

A total of 24 vine samples (*Vitis vinifera* L. var. Pinot noir clone 115) were used in the experiment. All the grapes were harvested by hand at 6 weeks post-veraison.

### 2.2. UV-B Treatment

Two UV-B treatments were applied to potted vines chosen for their similar leaf areas and crop loads. The vines were uniformly irrigated under normal conditions in a glasshouse. All treatments were applied from veraison to harvest.

(1) UV-B control treatment (−UV): 12 vines were exposed to normal sunlight and day length in the glasshouse. A UV filter was placed between the –UV chamber and +UV chamber to block UV. The environment in the glasshouse was maintained at 22 °C/18 °C, day/night, and a relative humidity of 70–80%.
(2) UV-B treatment (+UV): UV-B-313 UV fluorescent tubes (313, Q-Lab Company, Westlake, OH, USA) were supported above the 12 vines. Fluence rates of UV-B (280–313 nm) were measured by a UV-B Biometer model 501 radiometer (Solar Light Company, Glenside, PA, USA). The environment in the glasshouse was the same as for the control. In the UV area, the intensity of UV-B was maintained at UVI-6 for 8 h/d (9:00–17:00).

### 2.3. Water Treatment

Two water treatments were applied within each UV-B treatment group (Table 1). Time domain reflectometry (TDR) (Hydrosense™, Campbell Scientific, Inc., Logan, UT, USA) was used to evaluate the percentage of substrate soil moisture for each pot. All treatments were applied between veraisonand harvest:

(1) Well-watered control treatment (+W): Six vines in each UV treatment were regularly irrigated. The soil volumetric water content was around 30%.
(2) Water-deficit treatment (−W): Six vines received less than 50% of the water used in the well-watered irrigation treatment. The soil volumetric water content was around 10%.

### 2.4. Micro-Vinification

Around 100 g amounts of frozen grapes from each experimental plot were transferred into a plastic bag. Once the grapes had thawed (at room temperature), they were crushed by hand. The must was transferred into a 350 mL coffee plunger and left overnight at 4 °C, and then moved into a 26 °C chamber. Once the crushed grapes warmed to chamber temperature, each container was inoculated with 0.064 g Maurivin PDM of Saccharomyces cerevisiae (manufacturer’s recommended rate). Ferments were monitored initially using a balance to monitor weight loss and, therefore, fermentation progress. The plungers were pushed down and then back up gently twice per day until fermentation was complete. When the weight of the ferments stopped changing, wine density was measured with a density meter (DMA 35 Portable Density Meter, Anton Paar), and then they were covered with CO_2_. The density measurement was repeated each day. The primary fermentation was completed when °Brix was below 1.0 twice. The plunger was pushed down by hand. The supernatant wine was decanted into 50 mL plastic bottles with 35 mg/L SO_2_ solution and stored at −20 °C until analysis [10]. 

### 2.5. Amino Acid Analysis of Grapes and Wine by HPLC

The method of amino acid analysis of grapes and wine used in this study was similar to that described by Gregan et al. [11]:

Fifteen whole frozen grape berries of each treatment were ground into powder in liquid nitrogen. A 2–3 g amount of frozen grape powder was prepared to obtain the supernatant. Wine samples were from 2 mL of the supernatant wines. An internal standard (ɤ-amino butyric acid) was added to a final concentration of 100 µmol/L. 

Each sample was analysed using a Hewlett-Packard Agilent 1100 series HPLC system with a 250 × 4.6 mm, 5 µm prodigy column (Phenomenex). O-phthaldialdehyde and 9-fluorenylmethyl chloroformate were applied as fluorescence derivatives for primary amino acids and proline, respectively. A fluorescence detector with an excitation of 335 nm and emission of 440 nm was employed. The detector was switched into excitation 260 nm and emission 315 nm at 25 min to test proline. 

Two solvents were utilized in this method: solvent A (0.01 mol/L Na_2_HPO_4_ with 0.8% THF, adjusted to pH 7.5 with H_3_PO_4_) and solvent B (20% solvent A, 40% methanol, 40% acetonitrile). Gradient: 0 min, 0% B; 14 min, 40% B; 22 min, 55% B; 27 min, 100% B; 35 min, 100% B; 36 min, 0% B; with a flow rate of 1 mL/min. Chemstation (Agilent) chromatography data system was used for data analysis.

### 2.6. General Wine Parameters

pH was measured by using a calibrated pH electrode (SP-701, Suntex Instruments Co., Ltd., Taipei, Taiwan). A 10 mL volume wine sample was placed into an 80 mL flask. Then, the titratable acidity (TA) was measured by titration to pH 8.2 by standardised NaOH solution, following the method described by Iland P [12].

### 2.7. Wine Volatile Compound Analysis by GC-MS

Analysis of esters, alcohols, and aldehyde followed that described by Tomasino et al. [13]. Room-temperature sample wines were pipetted into 13 mL amber glass vials. The pH of dearomatized wine was adjusted to 3.5 [14]. All samples were kept at 8 °C before injection into a stack cooler attached to the Combi-Pal autosampler (CTC-Analytics, Zwingen, Switzerland). All wine samples were analysed on a Shimadzu QP2010 GC-MS (Shimadzu Scientific Instruments, Kyoto, Japan) induplicate in a randomised order.

### 2.8. Statistical Analysis

Statistical analysis was undertaken using IBM SPSS Statistics 22. The data were subjected to two-factor analyses (ANOVA) to partition the variance into the main effects and the interaction among them. In the case of significant interactions among factors, treatments were compared using the least significant difference (LSD) at the 5% level (*p* < 0.05).The principal component analysis (PCA) was performed through MiniTAB16 (Minitab, LLC, State College, PA, USA).

## 3. Result

### 3.1. Grape and Wine Amino Acids

There were no significant differences in °Brix or TA between treatments (Table 2), except for pH. Only UV-B (3.69 in +UV+W and 3.66 in +UV−W) significantly decreased pH compared with no UV-B treatments (3.76 in each of −UV+W and −UV−W). 

As shown in Table 2, total free amino acids and almost all the individual amino acids concentrations showed a decrease (25–84%)with increased UV-B.There was no consistent effects of water deficit on the concentrations of individual amino acids, and no statistically significant effect on total free amino acids concentration. 

Neither wine TA nor pH were significantly affected by UV-B exposure or water deficit (Table 3). The concentrations of amino acids in wines were different across treatments. UV-B exposure significantly decreased (by 41–93%) the concentrations of total amino acids and most individual amino acids groups as well: α-ketoglutarate group (Glu, Gln, and His), aromatic amino acid (Phe), pyruvate family (Leu, Val, and Ala), aspartate group (Asp, Thr, and Met), and 3-phosphoglycerate family (Ser, Cys, and Gly). Water deficit treatment had significant effects on final wine amino acids. Asp, Glu, Asn, and Ser were increased by reduced irrigation by 56–90%.

**Figure 1:**
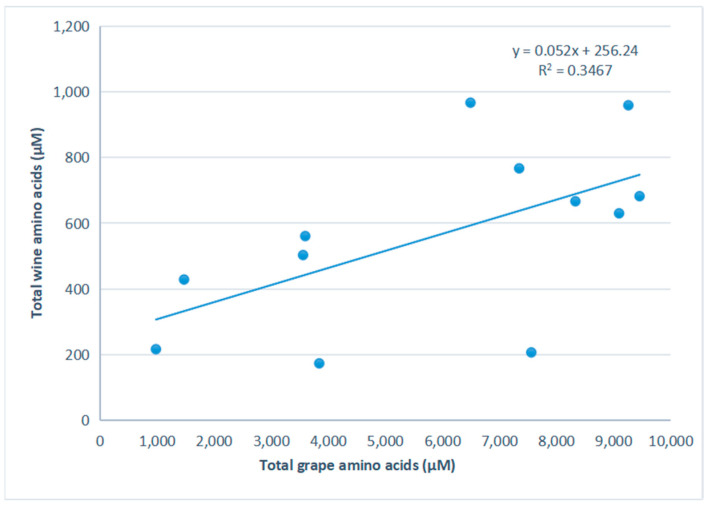
Relationship of total amino acids between grapes and wines.

The concentrations of total and individual amino acids in wines were significantly lower than those in grapes. In this study, we found a weaker correlation (R^2^ = 0.3467) between grape and wine amino acids (Figure 1); however, this relationship was highly influenced by the lower three points, which were similar in wine concentrations but significantly different in grape concentrations.

### 3.2. Amino Acid Consumption

**Figure 2:**
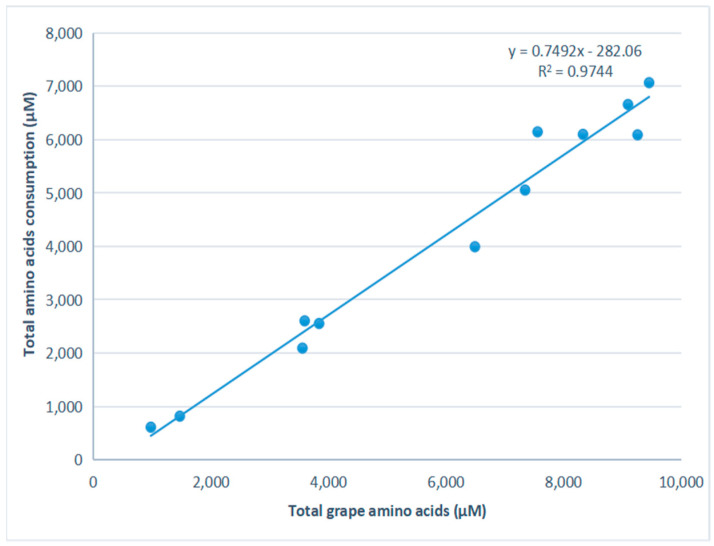
Relationship between initial concentration of amino acids and consumed amino acids.

Amino acid consumption was defined as differences between amino acid concentrations in the initial grapes and their amounts in final wines. The consumption of amino acids was significantly and positively correlated (R^2^ = 0.9744) with the concentration of initial grape amino acids (Figure 2).

The quantity of consumed amino acids was calculated from starting grapes and final wines (Table 4). All (100%) of Arg, 90–99% of Ala, and 82–98% of Asp were consumed by yeast. The proportions of consumed Glu, Thr, and Ser were high, at 84–95%, 85–96%, and 91–98%, respectively. The aromatic amino acid Trp was also totally consumed, independently of the number of amino acids in the must. The pyruvate amino acids, Val and Leu, were 70–92% and 56–97% consumed, respectively. Interestingly, Cys showed a negative consumption rate.

### 3.3. Wine Aroma

Table 5 listed the concentrations of aroma compounds in Pinot noir wines. UV exposure interaction with water deficit led to a lower concentration of linalool (10.58 μg/L) and a higher concentration of citronellol (11.46 μg/L) in wines compared to the control treatment. With regard to well-watered treatments, the concentrations of linalool, geraniol, and citronellol were affected by water deficit. The content of linalool and geraniol were decreased (by 29% and 34%, respectively) by water deficit without UV-B, while citronellol was increased (by 23%).

UV treatment did not influence the concentration of β-ionone, but it decreased the content of β-damascenone (27%). As for water deficit, both β-damascenone (22%) and β-ionone (15%) were increased by reduced irrigation. In addition, UV-B together with water deficit influenced β-damascenone concentration, where the highest concentration can be found in −UV−W treatment and the lowest level in +UV+W.

UV radiation had effects on phenylethyl alcohol in this study. In comparison to the UV exclusion treatment, +UV decreased phenylethyl alcohol by 8%. The level of isoamyl alcohol was not affected by UV treatment. Higher concentrations of isoamyl alcohol and phenylethyl alcohol were observed in wines from the water deficit treatment.

UV radiation had effects on the concentrations of hexanoic acids. The concentration of hexanoic acid (straight-chain fatty acids) in +UV treatment was19% lower than that in −UV treatment under reduced irrigation. Water deficit did not influence the level of hexanoic acids.

The UV treatment-induced changes of wine ester concentrations were as follows: Isoamyl acetate and ethyl acetate were decreased by increased UV-B under water deficit, by 34% and 50%, respectively. UV exposure treatment decreased ethyl esters of straight-chain fatty acids. Under water deficit, UV exposure significantly decreased the concentration of ethyl butanoate, ethyl hexanoate, and ethyl decanoate by 20–30%. Ethyl esters of branched-chain fatty acids, such as ethyl isobutyrate and ethyl isovalerate, were decreased by UV+ treatment by 5–42%. The aromatic esters, ethyl cinnamate and ethyl hydroxycinnamate, were not affected by UV radiation.

The concentration of isoamyl acetate was increased by water deficit, which was consistent with the trend of isoamyl alcohol. Ethyl esters of straight-chain fatty acids were affected by water deficit. The concentrations of ethyl acetate, ethyl butanoate, ethyl hexanoate, ethyl decanoate, and ethyl pentanoate were increased by reduced irrigation by 9–51% (under the UV exclusion conditions). Ethyl esters of branched fatty acids, such as ethyl isobutyrate and ethyl isovalerate, were increased by limited irrigation as well. In addition, reduced irrigation significantly decreased the concentration of phenylethyl acetate (9%) and ethyl cinnamate (13%). Additionally, water deficit interacting with UV-B exclusion increased the promotional effects on the concentrations of isoamyl and ethyl acetate, ethyl octanoate, and ethyl hexanoate; their concentrations were the highest in −UV−W and the lowest in +UV+W treatments. Effects of water deficit were opposite to the effects of UV-B exclusion, leading to similar results from +UV−W and −UV+W. 

### 3.4. PCA

**Figure 3:**
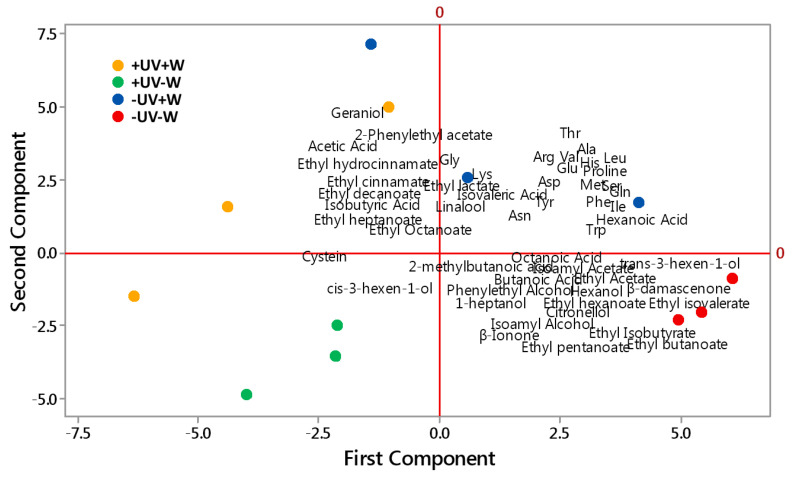
Principal component analysis of wine aroma compounds and amino acids.

There were four groups sortedby treatment, corresponding to +UV+W, +UV−W, −UV+W, and −UV−W (Figure 3). Clear separation was evident, with +UV−W located in the lower left quadrant, –UV−W in the lower right quadrant, +UV+W in the upper left quadrant, and −UV+W in the upper right quadrant.

The first component was positively correlated with hexanoic acid, butanoic acid, isovaleric acid, octanoic acid, 2-methylbutanoic acid, isoamyl acetate, ethyl acetate, ethyl hexanoate, isoamyl alcohol, phenylethyl alcohol, hexanol, 1-heptanol, trans-3-hexen-1-ol, ethyl isovalerate, ethyl isobutyrate, ethyl pentanoate, ethyl octanoate, ethyl butanoate, linalool, citronellol, β-damascenone, β-Ionone, Asp, Glu, Asn, Ser, Gln, His, Gly, Thr, Arg, Ala, Tyr, Val, Met, Trp, Phe, Ile, and Lys, whilst it was negatively correlated with ethyl cinnamate, ethyl hydrocinnamate, geraniol, ethyl decanoate, ethyl octanoate and ethyl heptanoate, phenylethyl acetate, cis-3-hexen-1-ol isobutyric acid, and acetic acid. 

The second component was negatively correlated with Cys, cis-3-hexen-1-ol, butanoic acid, octanoic acid, 2-methylbutanoic acid, ethyl pentanoate, ethyl isobutyrate, ethyl isovalerate, ethyl acetate, ethyl butanoate, ethyl hexanoate, 1-heptanol, hexanol, isoamyl acetate, isoamyl alcohol, phenylethyl alcohol, trans-3-hexen-1-ol, citronellol, β-damascenone, and β-Ionone, whilst it was positively correlated with almost all amino acids, ethyl octanate, ethyl cinnamate, ethyl hydrocinnamate, geraniol, phenylethyl acetate, linalool, hexanoic acid, isobutyric acid, acetic acid, ethyl heptanoate, and ethyl acetate.

## 4. Discussion

### 4.1. The Alteration of Amino Acids 

The concentrations of total and individual amino acids in wines were lower than that in grapes. Arias-Gil et al. [15] altered must amino acid content via addition of amino acids and found that the concentration of wine amino acids was directly proportional to the concentrations of must amino acids (R^2^ = 0.999). However, a weaker correlation was found in this study, which was highly influenced by three low amino acids points. All three points were similar in wine concentrations but significantly different in grape concentrations. This might be because wine nitrogen was not increased with must amino acids when the yeast assimilable nitrogen levels were similar in the wine, independently of must amino acid concentration [16].

### 4.2. Amino Acid Consumption

During fermentation, yeast utilized a great quantity of amino acids. The consumption of amino acids in this study was significantly and positively correlated with the concentration of initial grape amino acids. This result was consistent with previous findings [17].

Arg, Ala, Asp, Glu, Thr, and Ser were consumed in high proportions, likely because these forms of amino acids were preferred nitrogen sources for wine yeast [2]. The aromatic amino acid Trp was also totally consumed, independently of the amount of must amino acids; this result is consistent with that of Arias-Gil et al. [15]. Most of the branched amino acids, Val and Leu, were consumed during alcoholic fermentation. Interestingly, Cys consumption was negative, which meant its concentration increased during fermentation. This result was because the sulfate reduction sequence (SRS) pathway can transfer HS- to form methionine and Cys [18].

### 4.3. Changing Wine Aroma Compounds 

#### 4.3.1. Terpenes

Monoterpenes existed in free and odourless glycosidically bound forms in berries. During fermentation, yeast can release bound forms into odour-contributing forms in wines [19]. UV exposure interaction with water deficit decreased linalool concentration and increased citronellol in this study. This may be because the linalool synthesis enzyme was sensitive to sunlight. The accumulation of bound forms of terpenes can be mediated by exposure to the sun [20]. Linalool can be transformed into geraniol, nerol, and further into citronellol through acid-catalysed rearrangements [21]. Song et al. [14] found that UV transmitting treatments significantly increased the concentrations of nerol (37%) and geraniol (17%) compared to UV exclusion treatments. This might be due to linalool being increased by UV and then transformed into geraniol and nerol.

The concentrations of linalool, geraniol, and citronellol were affected by water deficit. Wang et al. [22] found that the accumulation rate of bound terpenes in Viognier grapes was reduced by irrigation. This fact could be related to abundant water supply increasing growth and yield, resulting in a delay in ripening, while water deficit generally had positive effects on grape ripening and phenolic compound accumulation. 

#### 4.3.2. C_13_-Norisoprenoids

UV treatment did not influence the concentration of β-ionone, but it decreased β-damascenone. This could be due to a decrease in berry maturity under the UV-B treatment (Table 5). It has been reported that higher maturity levels reflected higher levels of bound terpenols and C_13_-norisoprenoids [23]. However, UV radiation has been reported to increase β-damascenone in Riesling wine [24]. Hernandez-Orte et al. [25] indicated that yeast enzymes could affect the formation of C_13_-norisoprenoids during fermentation. Although the relationship between β-damascenone and amino acids can be seen in the study (Supplementary Table S1), the reduction in β-damascenone might be explained by the fact that the amino acid level of must was decreased by UV-B, possibly resulting in an indirect effect on β-damascenone formation.

Water deficits increased both β-damascenone and β-ionone, suggesting that water deficit affected the degradation of carotenoid contents of grapes, thereby possibly increasing C_13_-norisoprenoids [26]. This finding was important because a higher concentration of β-damascenone could contribute towards the sensory perception of floral, sweet, and cooked apple in Pinot noir wines [14]. In addition, the highest concentration of β-damascenone can be found in the−UV−W treatment, with the lowest level in the +UV+W treatment. This might be due to reduced irrigation strengthening the promotional influence of UV exclusion on β-damascenone accumulation.

#### 4.3.3. Alcohols

The reduction in phenylethyl alcohol concentration under UV-B was a result of the decrease in concentrations of amino acids [19]. Phenylethyl alcohol was not correlated with amino acids in this study (Supplementary Table S1), while Song et al. [14] indicated that UV treatment significantly increased phenylethyl alcohol. Higher alcohols were produced from amino acids by the Ehrlich pathway or from sugar by glycolysis during alcoholic fermentation [7]. However, there was no correlation between higher alcohols and their related amino acids in the study. It was possible that the higher alcohols were mainly produced from glycolysis via a biosynthesis pathway, not the Ehrlich pathway [27]. However, the level of isoamyl alcohol was not affected by UV treatment, which was in agreement with one previous study [14].

Water deficit increased the concentrations of isoamyl alcohol and phenylethyl alcohol. There were significant correlations between isoamyl alcoholand some and individual amino acids (Supplementary Table S1), which were affected by water deficit. The level of irrigation may have effects on the amount of the amino acids involved in thebiosynthesis of isoamyl alcohol. Moreover, limiting irrigation could increase the must nitrogen content and affect yeast fermentation [28]. Surplus alpha-keto acids can only be excreted as higher alcohols for a low nitrogen content of must [27]. Higher alcohols were regarded as a positive factor for the desirable complexity of wine below 300 mg/L. Above 400 mg/L, however, the fusel alcohols can contribute to negative aromas in wine [29].

#### 4.3.4. Fatty Acids

In this study, there were some significant correlations between medium-chain fatty acids and several individual amino acids (Supplementary Table S1), which could suggest that amino acids have effects on yeast cells in adapting their membrane fatty acids and increasing yeast growth and glycolysis rate [30]. Although it has been reported that straight-chain fatty acids were affected by the availability of unsaturated fatty acids and fermentation conditions, it was suggested that must nitrogen content could affect fatty acids metabolism as well [31]. Cheng et al. [31] observed that the formation of medium-chain fatty acids was increased by the addition of branched amino acids and Phe in must. Reduced UV-B increased the level of hexanoic acids, which might be because decreased UV-B radiation could increase the amino acids level in the must, subsequently affecting the formation of this medium-chain fatty acid. These increases in fatty acids can contribute to a fresh flavour in wine, with fatty, cheese, rancid, and fresh notes when their level is above 20 mg/L. At low concentrations, they contribute to the complexity of wine aroma [32].

#### 4.3.5. Esters

Isoamyl acetate was decreased under UV-B exposure, which was consistent with Wang et al. [33]. Acetate esters were increased by addition of branched amino acids and Phe. This might because UV-B decreased the level of amino acids involved in acetate ester formation (the relationship between Leu and isoamyl acetate). It indicated Leu might involve in isoamyl acetate formation. Although the concentration of ethyl acetate was decreased under UV-B radiation, Song et al. [14] reported there was no effect of UV-B on this ester. The reduction in ethyl acetate might be due to decreases in general amino acid concentrations under UV-B. The consumption of total amino acid concentration generally affected yeast fermentation to support the relationship between isoamyl acetate, ethyl acetate and amino acid concentrations. Paul et al. [34] indicated that nitrogen availability could affect expression of the ATF1 gene for encoding alcohol acetyltransferase enzymes. 

UV exposure decreased ethyl esters of straight-chain fatty acids in accordance with the results of Song et al. [14]. UV exposure also significantly reduced the concentration of ethyl butanoate, ethyl hexanoate, and ethyl decanoate. This was consistent with the close relationship between medium-chain fatty acids and ethyl esters of medium-chain fatty acids in the study. Ethyl esters of branched-chain fatty acids, such as ethyl isobutyrate and ethyl isovalerate, were decreased by +UV treatment. Song et al. [14] also observed a decrease in ethyl esters of branched-chain fatty acids, which could be related to UV-B-induced effects of amino acids or amino acids derivatives. These ethyl esters of fatty acid acetates can be important because they contribute global “fruity” aroma and complexity to wine [35].

The aromatic esters, ethyl cinnamate and ethyl hydroxycinnamate were not affected by UV radiation. Song et al. [14] found the concentration of phenylethyl acetate stayed constant under UV treatments, but the concentration of ethyl cinnamate was decreased (26%) by +UV treatment. These ethyl esters of fatty acids acetates contribute global “fruity” aroma and complexity to wines [34].

The concentration of isoamyl acetate was increased by water deficit in consistent with the trend of isoamyl alcohol. Ethyl esters of straight-chain and branched fatty acids were affected by water deficit. The increase in esters could be supported by water deficit-induced the increase in grape amino acids. In addition, water stress significantly decreased the concentration of phenylethyl acetate and ethyl cinnamate. Drought may promote the synthesis of volatile compounds and their precursors in the grapes, which combined with the differences in vine vigor and vegetative growth. Drought, also, may have other effects on grape maturity, leading to variations of a range of aroma compounds in wines [36]. 

### 4.4. PCA 

In general, wines from −UV−W treatment grapes tended to have a more diverse set of aroma compounds compared with other treatments, potentially resulting in more fruity and floral sensory characteristics. PCA analysis showed that the treatment effects were distinguishable, demonstrating the influence of environmental factors or viticulture management (such as UV-B radiation and deficit irrigation) on grape amino acids profiles. This translates to changes in wine amino acid profiles and wine aroma-active compounds.

## 5. Conclusions

To conclude, the initial grape amino acid concentrations were altered by UV-B and irrigation treatments. Amino acids are the major nitrogen source for yeast during alcoholic fermentation and act as precursors of fermentation-derived aroma compounds; therefore, the changes in amino acids are ultimately translated into wine aroma compounds. This study highlights the amino acid consumption between grapes and wines during the fermentation, potentially leading to a method of predicting the quantity of key wine aroma parameters. Although, there are still several uncertainties around this exact relationship, it may provide a practical way to increase the volatile compounds of fruit through irrigation management and UV exposure. It may contribute to change notes with the anticipated specific characteristics in the final wines.

## Figures and Tables

**Table 1:** Treatments of glasshouse trial.

	UV-B Radiation	UV-B Exclusion
Well water	+UV+W (6 vines)	−UV+W (6 vines)
Water stress	+UV−W (6 vines)	−UV−W (6 vines)

**Table 2:** Grape parameters and amino acids at harvest (μM).

	Treatment				*p* Value		
	+UV+W	+UV−W	−UV+W (Control)	−UV−W	UV	W	UV*Water
°Brix	21.0	19.1	21.0	22.0	n.s.	n.s.	n.s.
TA	5.4	5.4	6.6	6.4	n.s.	n.s.	n.s.
pH	3.69	3.66	3.76	3.76	0.04	n.s.	n.s.
*α-ketoglutarate*							
Proline (Pro)	891	608	1765	1869	0.00	n.s.	n.s.
Argnine (Arg)	976	494	2176	1324	0.00	0.00	n.s.
Glutamate (Glu)	331	253	532	485	0.00	0.01	n.s.
Glutamine (Gln)	158	127	380	417	0.00	n.s.	n.s.
Histidine (His)	84	72	174	150	0.00	n.s.	n.s.
*Shikimate (Aromatic)*							
Phenylalanine (Phe)	34	26	70	101	0.00	0.01	0.00
Tryptophane (Trp)	35	52	59	69	0.00	0.02	n.s.
Tyrosine (Tyr)	4	0	9	22	0.00	0.02	0.00
*Pyruvate*							
Leucine (Leu)	56	46	150	163	0.00	n.s.	n.s.
Valine (Val)	93	95	203	178	0.00	n.s.	n.s.
Alanine (Ala)	692	490	1532	1238	0.00	0.04	n.s.
*Aspartate*							
Aspartic acid (Asp)	174	99	363	309	0.00	0.01	n.s.
Asparagine (Asn)	5	15	49	60	0.00	0.00	n.s.
Threonine (Thr)	283	206	713	540	0.00	0.02	n.s.
Isoleucine (Ile)	27	39	86	118	0.00	0.02	n.s.
Methionine (Met)	11	5	23	31	0.00	n.s.	0.05
Lysine (Lys)	17	28	55	39	0.00	n.s.	0.00
*3-phosphoglycerate*							
Cysteine (Cys)	N.A	N.A	N.A	N.A	N.A	N.A	N.A
Serine (Ser)	242	198	571	517	0.00	n.s.	n.s.
Glycine (Gly)	11	20	49	67	0.02	0.01	n.s.
Total	4124	2873	8959	7697	0.00	n.s.	n.s.

Data presented as the mean of three replicates from harvest in 2015–2016, with *p*-values for statistical significance comparing the different treatments according to two-factor ANOVA and LSD test at the 5% level. N.A, not available; *p*UV, UV effects averaged across water treatments; *p*water, water effects averaged across UV treatments; *p*UV*water, water effects depend on UV treatments and UV effects depend on water treatments; n.s., no significant difference; +W, well-watered, −W, water deficit; +UV, UV-B radiation, −UV, normal light.

**Table 3:** Wine parameters and amino acids (μM).

	Treatment				*p* Value		
	+UV+W	+UV−W	−UV+W (control)	−UV−W	UV	W	UV*Water
TA	7.1	5.9	7.2	6.0	n.s.	n.s.	n.s.
pH	4.4	4.0	4.4	4.7	n.s.	n.s.	n.s.
*α-ketoglutarate*							
Pro	69.8	69.9	72.8	114.9	0.00	n.s.	n.s.
Arg	4.4	3.2	2.5	4.6	0.03	n.s.	n.s.
Glu	17.7	40.3	49.6	67.2	0.00	0.00	n.s.
Gln	2.8	18.5	19.0	24.5	0.00	n.s.	n.s.
His	3.2	9.3	10.9	14.5	0.03	n.s.	n.s.
*Shikimate (Aromatic)*							
Phe	1.2	10.5	16.4	22.7	0.02	n.s.	n.s.
Trp	0.8	1.2	1.4	2.2	n.s.	n.s.	n.s.
Tyr	1.4	7.5	11.6	16.8	n.s.	n.s.	0.02
*Pyruvate*							
Leu	1.9	20.4	30.3	45.5	0.01	n.s.	n.s.
Val	7.2	27.8	36.0	53.3	0.02	n.s.	n.s.
Ala	7.3	48.4	77.1	119.9	0.01	n.s.	n.s.
*Aspartate*							
Asp	3.4	17.4	24.8	32.5	0.00	0.00	0.02
Asn	3.6	37.1	50.1	62.7	0.00	0.00	0.04
Thr	11.1	31.6	35.2	52.1	0.00	n.s.	n.s.
Ile	1.5	13.1	18.9	29.3	0.01	n.s.	n.s.
Met	0.6	3.4	4.8	7.3	0.02	n.s.	n.s.
Lys	4.2	15.9	24.4	32.6	n.s.	n.s.	n.s.
*3-phosphoglycerate*							
Cys	47.7	73.4	102.0	86.1	0.00	n.s.	0.00
Ser	5.4	17.1	21.8	33.5	0.00	0.00	n.s.
Gly	3.8	31.7	50.1	75.2	n.s.	n.s.	n.s.
Total	199	497.7	659.7	897.4	0.00	n.s.	n.s.

Data presented as the mean of three replicates from harvest in 2015–2016, with *p*-values for statistical significance comparing the different treatments according to two-factor ANOVA and LSD test at the 5% level. *p*UV, UV effects averaged across water treatments; *p*water, water effects averaged across UV treatments; *p*UV*water, water effects depend on UV treatments and UV effects depend on water treatments; n.s., no significant difference; +W, well-watered, −W, water deficit; +UV, UV-B radiation, −UV, normal light.

**Table 4:** Amino acids consumption (μM).

	Treatment				*p* Value		
	+UV+W	+UV−W	−UV+W	−UV−W	UV	W	UV*Water
*a-ketoglutarate*							
Arg	972	491	2173	1320	0.03	n.s.	n.s.
Glu	314	213	482	418	0.00	n.s.	n.s.
Gln	155	109	361	393	0.00	n.s.	n.s.
His	81	62	163	136	0.03	n.s.	n.s.
*Shikimate (Aromatic)*							
Phe	33	16	53	78	0.02	n.s.	n.s.
Trp	34	51	57	67	n.s.	n.s.	n.s.
Tyr	2	−7	−2	5	n.s.	n.s.	0.02
*Pyruvate*							
Leu	54	26	120	118	0.01	n.s.	n.s.
Val	86	67	167	125	0.02	n.s.	n.s.
Ala	685	442	1455	1118	0.01	n.s.	n.s.
*Aspartate*							
Asp	171	82	338	277	0.02	n.s.	n.s.
Asn	1	−22	−1	−3	n.s.	n.s.	n.s.
Thr	272	175	678	488	0.00	n.s.	n.s.
Ile	26	25	67	88	0.01	n.s.	n.s.
Met	11	1	19	23	0.02	n.s.	n.s.
Lys	13	12	31	7	n.s.	n.s.	n.s.
*3-phosphoglycerate*							
Cys	−48	−73	−102	−86	0.00	n.s.	0.00
Ser	236	181	549	483	0.00	n.s.	n.s.
Gly	7	−11	−1	−8	n.s.	n.s.	n.s.
Total	3104	1838	6608	5045	0.00	n.s.	n.s.

Data presented as the mean of three replicates from harvest in 2015–2016, with *p*-values for statistical significance comparing the different treatments according to two-factor ANOVA and LSD test at the 5% level. *p*UV, UV effects averaged across water treatments; *p*water, water effects averaged across UV treatments; *p*UV*water, water effects depend on UV treatments and UV effects depend on water treatments; n.s, no significant difference; +W, well-watered, −W, water deficit; +UV, UV-B radiation, −UV, normal light.

**Table 5:** The concentrations of aroma compounds in experimental wines (μg/L).

	Treatment				*p* Value		
	+UV+W	+UV−W	−UV+W	−UV−W	UV	W	UV*water
Ethyl isobutyrate	9.89	15.85	12.92	19.08	0.03	0.00	n.s.
Ethyl butanoate	12.06	22.48	15.88	32.51	0.02	0.00	n.s.
Ethyl isovalerate	0.28	0.45	0.38	0.64	0.00	0.00	n.s.
Isoamyl acetate	147	115	115	174	0.05	0.05	0.00
Ethyl pentanoate	0.35	0.4	0.36	0.43	n.s.	0.00	n.s.
Isoamyl alcohol	131,416	160,616	128,260	194,095	n.s.	0.01	n.s.
Ethyl hexanoate	67.13	71.06	66.04	90.71	0.04	0.01	0.03
Hexyl acetate	0.99	0.73	0.8	0.94	n.s.	n.s.	0.00
Hexanol	812	833	798	1120	n.s.	n.s.	n.s.
trans-3-hexen-1-ol	16.14	15.73	17.46	21.33	n.s.	n.s.	n.s.
Ethyl heptanoate	0.52	0.42	0.46	0.41	n.s.	n.s.	n.s.
cis-3-Hexen-1-ol	17.65	24.79	18.72	16.15	0.04	n.s.	0.02
1-Heptanol	15.73	16.22	14.89	17.25	n.s.	n.s.	n.s.
Ethyl octanoate	252	188	190	240	n.s.	n.s.	0.00
Benzaldehyde	12.97	10.53	13.84	11.96	n.s.	0.03	n.s.
Ethyl decanoate	394	235	266	292	0.04	0.00	0.00
Phenylethyl alcohol	39223	45285	44826	49417	0.01	0.00	n.s.
Ethyl acetate	44,458	39,546	42,704	79,278	0.00	0.00	0.00
Acetic acid	2,727,645	1,265,985	2,040,245	1,499,374	n.s.	0.02	n.s.
Isobutyric acid	3498	3864	3672	3009	n.s.	n.s.	n.s.
Butanoic acid	825	1038	1008	1025	n.s.	0.04	n.s.
Isovaleric acid	302	299	355	365	n.s.	n.s.	n.s.
2-Methylbutanoic acid	374	452	413	419	n.s.	n.s.	n.s.
Hexanoic acid	672	672	785	826	0.00	n.s.	n.s.
Octanoic acid	464	378	441	565	n.s.	n.s.	n.s.
Linalool	9.48	10.58	13.9	9.83	0.01	0.03	0.00
Citronellol	8.16	11.46	10.28	13.29	0.05	0.01	n.s.
Phenylethyl acetate	22.68	14.94	21.68	19.82	n.s.	n.s.	n.s.
*β*-Damascenone	4.71	4.76	4.94	6.36	0.00	0.01	0.02
Geraniol	3.81	3.06	3.93	2.92	n.s.	0.00	n.s.
Ethyl hydrocinnamate	0.07	0.05	0.07	0.05	n.s.	n.s.	n.s.
β-Ionone	0.45	0.5	0.43	0.5	n.s.	n.s.	n.s.
Ethyl cinnamate	0.53	0.19	0.45	0.22	n.s.	0.03	n.s.

Data presented as the mean of three replicates from harvest in 2015–2016, with *p*-values for statistical significance comparing the different treatments according to Two-factor ANOVA and LSD test at the 5% level. *P_UV_*, UV effects averaged across water treatments; *P_water_*, water effects averaged across UV treatments; P_UV*water_, water effects depend on UV treatments and UV effects depend on water treatments; n.s., no significant difference; +W, well-watered, −W, water deficit; +UV, UV-B radiation, −UV, normal light.

## Data Availability

All data analyzed or generated during this study are available withinthe manuscript and can be requested from the corresponding author.

## Supplementary Materials

The following supporting information can be downloaded at: https://www.mdpi.com/article/10.3390/foods11091336/s1, Table S1: The correlation between the concentrations of grape-derived aroma compounds and the consumption of individual amino acids.
