# Supplementary material for: Effects of UV-B and Water Deficit on Aroma Precursors in Grapes and Flavor Release during Wine Micro-Vinification and Consumption
Source: Foods. 2022 May 4;11(9):1336. doi: 10.3390/foods11091336 (PMC9103088; doi:10.3390/foods11091336)
Supplement: Supplementary file 1 [file foods-11-01336-s001.zip › foods-1681262 - Supplementary.pdf]

foods

MDPI

Supplementary Table 1*The correlation between the concentrations of grape-derived aroma compounds and the consumption of individual amino acids*

	Arg	Glu	Gln	His	Phe	Tyr	Trp	Leu	Val	Ile	Asp	Thr	Met	Lys	Ser	Gly	Asn	Ala
Acetic acid	n.s	.902*	.952**	.879*	n.s	n.s	.902*	.944**	.948**	.911*	n.s	n.s	.825*	n.s	.990*	n.s	n.s	n.s
Isobutyric acid	-.825*	n.s	n.s	n.s	-.902*	-.928**	n.s	-.827*	-.835*	n.s	n.s	-.932**	n.s	n.s	-.927*	n.s	n.s	-.956**
Butanoic acid	n.s	n.s	n.s	n.s	n.s	n.s	n.s	n.s	n.s	n.s	n.s	n.s	n.s	-.887*	n.s	-.943**	n.s	n.s
Isovaleric acid	n.s	-.911*	.840*	n.s	n.s	.814*	.860*	n.s	n.s	n.s	n.s	n.s	n.s	n.s	n.s	n.s	n.s	n.s
2-Methybutanoic acid	-.903*	n.s	n.s	-.937**	n.s	n.s	n.s	n.s	n.s	n.s	n.s	-.852*	n.s	n.s	n.s	n.s	n.s	-.845*
Hexanoic acid	n.s	.907*	.937**	n.s	.911*	.928**	.880*	.935**	.861*	.888*	n.s	.843*	.936**	n.s	.933**	n.s	n.s	.865*
Octanoic acid	.816*	n.s	n.s	n.s	.953**	.945**	n.s	.848*	n.s	n.s	n.s	.904*	.848*	n.s	.913*	n.s	n.s	.943**
Phenylethyl alco-hol	n.s	n.s	n.s	n.s	n.s	n.s	n.s	n.s	n.s	n.s	n.s	n.s	n.s	n.s	n.s	n.s	n.s	n.s
Isoamyl alcohol	0.867*	n.s	n.s	n.s	0.913*	0.984**	n.s	n.s	n.s	n.s	0.828*	0.953**	n.s	n.s	0.931**	n.s	n.s	0.962**
Ethyl isobutyrate	n.s	n.s	n.s	n.s	n.s	n.s	n.s	n.s	n.s	n.s	0.881*	n.s	n.s	n.s	n.s	n.s	n.s	n.s
Ethyl butanoate	n.s	0.955**	0.941**	n.s	0.864*	0.927**	0.906*	0.925*	0.853*	0.855*	n.s	0.885*	0.871*	n.s	0.965**	n.s	n.s	0.880*
Ethyl isovalerate	n.s	0.900*	0.857*	n.s	n.s	0.898*	0.877*	0.826*	n.s	n.s	0.873*	0.880*	n.s	n.s	0.935*	n.s	n.s	0.855*
Isoamyl acetate	0.837*	0.898*	0.881*	0.817*	0.899*	0.942**	n.s	0.904*	0.846*	n.s	0.835*	0.968**	0.823*	n.s	0.994**	n.s	n.s	0.957**
Ethyl pentanoate	n.s	n.s	n.s	n.s	n.s	n.s	n.s	n.s	n.s	n.s	n.s	n.s	n.s	n.s	n.s	n.s	n.s	n.s
Ethyl hexanoate	n.s	n.s	0.897*	n.s	0.886*	0.941**	0.875*	0.905*	0.880*	0.841*	n.s	0.887*	0.864*	n.s	0.952**	n.s	n.s	0.904*
Ethyl heptanoate	n.s	n.s	n.s	n.s	n.s	n.s	n.s	n.s	n.s	n.s	n.s	n.s	n.s	n.s	n.s	n.s	n.s	n.s
Ethyl octanoate	n.s	n.s	0.862*	n.s	0.907*	0.855*	n.s	0.880*	n.s	0.857*	n.s	n.s	0.953**	n.s	0.822**	n.s	n.s	n.s
Ethyl decanoate	n.s	0.854*	0.886*	n.s	0.880*	0.850*	0.800*	0.879*	n.s	0.868*	n.s	n.s	0.954**	n.s	0.815*	n.s	n.s	n.s
Ethyl acetate	n.s	0.941**	0.926**	n.s	0.896*	0.931**	0.859*	0.933**	0.863*	0.828*	n.s	0.933**	0.867*	n.s	0.988*	n.s	n.s	0.924**
Phenylethyl alco-hol	n.s	0.969**	0.911*	n.s	n.s	n.s	n.s	0.884*	n.s	0.833*	n.s	n.s	0.844*	n.s	0.822*	n.s	n.s	n.s
Linalool	n.s	n.s	n.s	n.s	n.s	-0.927**	n.s	n.s	n.s	n.s	-0.875*	-0.876*	n.s	n.s	n.s	n.s	n.s	-0.882*

Citronellol	n.s	0.848*	n.s	n.s	n.s	n.s	n.s	n.s	n.s	n.s	n.s	n.s	n.s	n.s	n.s	n.s	n.s	n.s
2-Phenylethyl acetate	n.s	0.969**	0.911*	n.s	n.s	n.s	n.s	0.884*	n.s	0.833*	n.s	n.s	0.844*	n.s	0.822*	n.s	n.s	n.s
β-Damascenone	n.s	0.989**	0.952**	n.s	0.816*	n.s	0.849*	0.932*	0.814*	0.878*	n.s	n.s	0.900*	n.s	0.884*	n.s	n.s	n.s
Geraniol	n.s	n.s	n.s	n.s	n.s	n.s	n.s	n.s	n.s	n.s	n.s	n.s	n.s	0.881*	n.s	n.s	n.s	n.s
Ethyl hydroxycinnamate	n.s	n.s	n.s	n.s	n.s	n.s	n.s	n.s	n.s	n.s	n.s	n.s	n.s	n.s	n.s	n.s	n.s	n.s
α-Ionone	n.s	n.s	n.s	n.s	n.s	n.s	n.s	n.s	n.s	n.s	n.s	n.s	n.s	n.s	n.s	n.s	n.s	n.s
Ethyl cinnamate	n.s	n.s	n.s	n.s	n.s	n.s	n.s	n.s	n.s	n.s	n.s	n.s	n.s	n.s	n.s	n.s	n.s	n.s

* Correlation is significant at the 0.05 level (2-tailed).

** Correlation is significant at the 0.01 level (2-tailed)
